# Reduction of Abeta amyloid pathology in APPPS1 transgenic mice in the absence of gut microbiota

**DOI:** 10.1038/srep41802

**Published:** 2017-02-08

**Authors:** T. Harach, N. Marungruang, N. Duthilleul, V. Cheatham, K. D. Mc Coy, G. Frisoni, J. J. Neher, F. Fåk, M. Jucker, T. Lasser, T. Bolmont

**Affiliations:** 1 Laboratoire d’Optique biomédicale, Institute of Microengineering, School of Engineering, Ecole Polytechnique Fédérale de Lausanne, CH1015 Lausanne, Switzerland; 2 Food for Health Science Centre, Lund University, Medicon Village, 22381 Lund, Sweden; 3 Mucosal Immunology Lab, Department of Clinical Research, University of Bern, Murtenstrasse, 35 CH - 3010 Bern, Switzerland; 4 Memory Clinic and LANVIE - Laboratory of Neuroimaging of Aging, University Hospitals and University of Geneva, Geneva, Switzerland; 5 German Centre for Neurodegenerative Diseases (DZNE), Tübingen, D-72076 Tübingen, Germany; 6 Department of Cellular Neurology, Hertie Institute for Clinical Brain Research, University of Tübingen, D-72076 Tübingen, Germany; 7 Stemedica International, Avenue de Sévelin 20, CH1004 Lausanne, Switzerland

## Abstract

Alzheimer’s disease is the most common form of dementia in the western world, however there is no cure available for this devastating neurodegenerative disorder. Despite clinical and experimental evidence implicating the intestinal microbiota in a number of brain disorders, its impact on Alzheimer’s disease is not known. To this end we sequenced bacterial 16S rRNA from fecal samples of Aβ precursor protein (APP) transgenic mouse model and found a remarkable shift in the gut microbiota as compared to non-transgenic wild-type mice. Subsequently we generated germ-free APP transgenic mice and found a drastic reduction of cerebral Aβ amyloid pathology when compared to control mice with intestinal microbiota. Importantly, colonization of germ-free APP transgenic mice with microbiota from conventionally-raised APP transgenic mice increased cerebral Aβ pathology, while colonization with microbiota from wild-type mice was less effective in increasing cerebral Aβ levels. Our results indicate a microbial involvement in the development of Abeta amyloid pathology, and suggest that microbiota may contribute to the development of neurodegenerative diseases.

Alzheimer’s disease (AD) is a severe and ever-growing socio-economic burden to western societies. According to the amyloid cascade hypothesis of AD pathogenesis, the aggregation and cerebral deposition of amyloid-β (Aβ) peptides into extracellular amyloid plaques is an early and critical event triggering a cascade of pathological incidents that finally lead to dementia^1^. Thus, arguing in favor of this hypothesis, the most rational strategy for an AD therapy would be to retard, halt and even reverse Aβ aggregation. However, despite all research efforts there is currently no treatment for AD, and currently approved therapies only provide symptomatic treatments for this disease. Numerous studies indicate that microbial communities represent an essential factor for many physiological processes including nutrition, inflammation, and protection against pathogens^2^,^3^. The microbial community is largely composed of bacteria that colonize all mucosal surfaces, with the highest bacterial densities found in the gastrointestinal tract. A growing body of clinical and experimental evidence suggests that gut microbiota may contribute to aging and influence brain disorders^4^. In particular, a new connection between gut microbiota and Parkinson’s disease has been reported in humans^5^. In mouse models, studies report a role for the microbiota in the modulation of stress-related behaviors relevant to psychiatric disorders^6^,^7^,^8^,^9^. Recent research has revealed that microbiota may impact the development of autism spectrum disorders (ASD) as *Bacteroides fragilis* improved defects in communicative and sensorimotor behaviors following maternal immune activation in mice, a mouse model exhibiting ASD features^10^. In a mouse model of multiple sclerosis, gut microbiota strongly contributes to pathology^11^. Recently, a study revealed an association of brain amyloidosis with pro-inflammatory gut bacteria of cognitively impaired patients^12^. Furthermore, a recent study showed that antibiotic-mediated perturbations in the gut microbiome modulates amyloid deposition in an AD mouse model^13^. While such findings strongly suggest that the gut microbiota may impact a wide range of brain disorders including AD, the impact of complete depletion of intestinal microbes on AD pathogenesis is unknown. To this end we evaluated the gut microbiota composition in a conventionally-raised AD mouse model^14^, and next generated a germ-free mouse model of cerebral β-amyloidosis to study the role of the gut microbiota.

## Results

### CONVR-APPPS1 mice display alterations in gut microbiota composition

**Figure 1:**
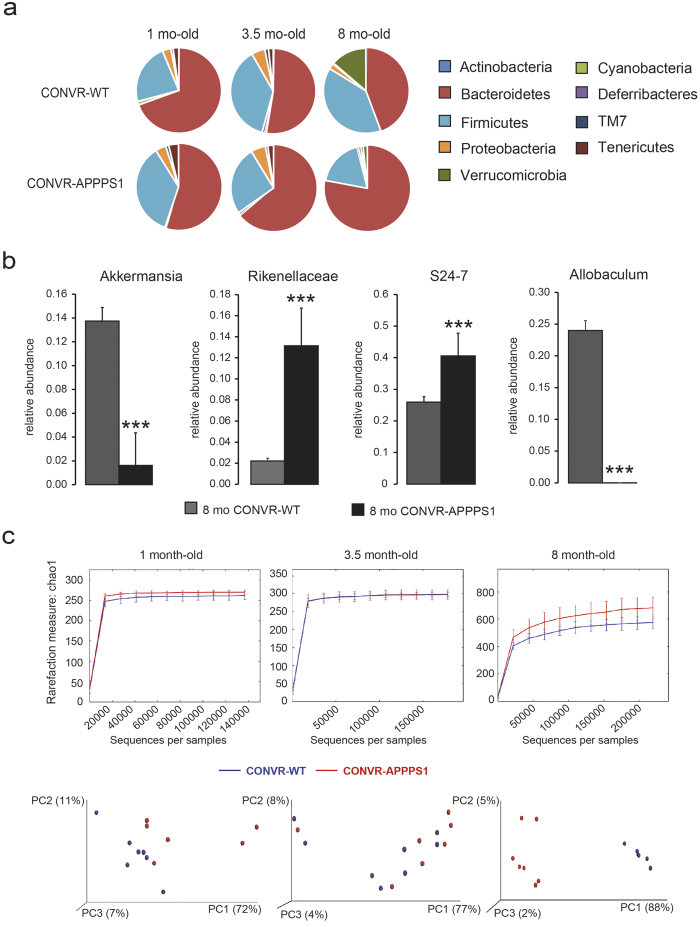
Comparison of the gut microbiota between conventionally raised (CONVR-) APPPS1 and wild type (WT) mice analyzed with multiple t-tests together with the Holm-Sidak method to correct for multiple comparisons. **(a)** Mean sequence relative abundance of gut microbial taxa at phylum level in CONVR-APPPS1 and WT mice, aged 1 month (n = 6 and n = 8 respectively), 3.5 months (n = 7 and n = 8) and 8 months (n = 6 and n = 7). Bacteroides, Firmicutes, Verrucomicrobia and Tenericutes were significantly different between 8 month-old APPPS1 and WT mice (p < 0.001), as well as Proteobacteria (p < 0.01) and Actinobacteria (p < 0.01). No significant differences were found in the younger mice. **(b)** At genus level, 4 microbial taxa with relative abundance >5% were significantly different between 8 month-old CONVR-APPPS1 and WT mice (***p < 0.001), while younger mice did not show any significant differences. **(c)** Rarefaction curves (α-diversity vs. sequencing effort) and weighted Unifrac PCoA plot to compare phylogenetic distance matrices of CONVR-APPPS1 and WT mice. Species richness was significantly increased in the 8 month-old APPPS1 mice (p < 0.05, observed species test in QIIME with FDR correction for multiple comparisons). Younger mice did not show significant differences in α-diversity. Clustering of CONVR-APPPS1 mice in the PCoA was significantly separated from the WT mice at 8 months of age (p < 0.001 for both the ANOSIM and the Adonis non-parametric test in QIIME). Experiments were performed three times, and data represent mean values of a representative experiment.

In a first set of experiments we studied the age-related changes in the intestinal microbiota in conventionally-raised transgenic APPPS1 mice (CONVR-APPPS1)^14^. CONVR-APPPS1 mice start to develop cerebral Aβ-deposition at 1.5 months of age and deposition increases thereafter^14^. We sequenced bacterial 16S rRNA genes extracted from fecal samples of pre-depositing 1 month-old and Aβ-depositing 3.5 and 8 month-old CONVR-APPPS1 and compared it to non-transgenic wild-type littermates (CONVR-WT). Results revealed major age-related shifts in the gut microbiota composition at both phylum and genus level in the CONVR-APPPS1 mice that were more pronounced than those in the CONVR-WT mice (Fig. 1a,b). At 8 month of age CONVR-APPPS1 mice displayed significant reductions in Firmicutes (p < 0.001), Verrucomicrobia (p < 0.001), Proteobacteria (p < 0.01) and Actinobacteria (p < 0.01) with a concurrent increase in Bacteroidetes (p < 0.001) and Tenericutes (p < 0.001) phyla as compared to 8 month-old CONVR-WT mice. Comparing high-abundant bacterial genera (relative abundance >5%), the 8 month-old CONVR-APPPS1 had significantly increased abundance of unclassified genera in *Rikenellaceae* and unclassified genus in *S24*-*7* (p < 0.001), while *Allobaculum* and *Akkermansia* (p < 0.001) were decreased as compared to CONVR-WT mice (Fig. 1b). An additional 10 low-abundant (<5%) genera differed significantly between CONVR-APPPS1 and CONVR-WT mice (data not shown). Furthermore, and surprisingly, α-diversity was significantly higher in the 8 month-old CONVR-APPPS1 compared to age-matched CONVR-WT mice (p < 0.05) (Fig. 1c). Analysis of β-diversity using weighted (Fig. 1c) and unweighted (data not shown) UniFrac showed a significant clustering of the 8 month-old mice depending on genotype (p < 0.001). Next we studied whether the differences in gut microbiota composition in CONVR-APPPS1 mice compared to CONVR-WT mice are the results of differences in metabolic parameters^15^. However, body weight and food intake did not differ between the groups, although cecal weight was significantly higher in the 8 month-old CONVR-APPPS1 mice as compared to the CONVR-WT mice (p < 0.05) (Fig. S1). Furthermore, there was no significant difference in caloric content of the diet before and after the autoclaving process (Fig. S1). Strikingly, however, in the 8 month-old CONVR-APPPS1 mice, the relative abundance of several bacterial genera was correlated with levels of Aβ42 in the brain (Fig. S2), suggesting a link between cerebral ß-amyloidosis and the gut microbiome.

### Generation of germ-free APPPS1 transgenic mice

To further study the link between the microbiome and cerebral ß-amyloidosis, we generated APPPS1 mice without gastro-intestinal microbiota, referred to axenic, or germ-free (GF)-APPPS1 mice. We then compared GF-APPPS1 mice with CONVR-APPPS1 at 1, 3, 5 and 8 months of age. Bacteriological evaluation of the GF-APPPS1 animals was regularly performed for their germ-free status by aerobic and anaerobic culture, DNA and gram staining of cecal content to detect uncultivable contamination. In addition, serological testing for known viruses and pathogens was performed periodically by ELISA, PCR or IFA tests (see methods for details). Although GF-APPPS1 mice were raised in flexible film isolators while CONVR-APPPS1 were raised in ventilated cages under SPF condition, all efforts were made to treat similarly the two groups (e.g., same autoclaved food, same handling, always n = 5/cage; see method).

### Aβ levels are reduced in GF-APPPS1 mice compared to CONVR-APPPS1 mice

**Figure 2:**
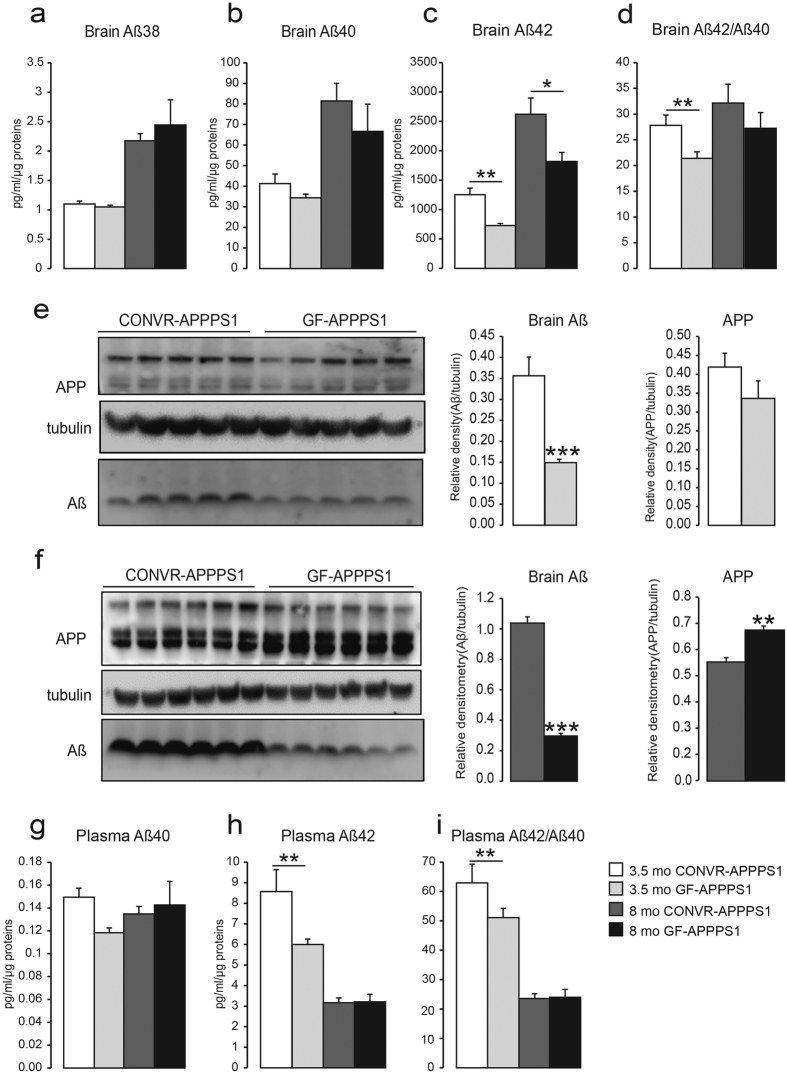
Reduction of Aβ levels in brain and blood of GF-APPPS1 transgenic mice. **(a**–**d)** Levels of Aβ38, Aβ40, Aβ42 and Aβ42/Aβ40 ratio measured by ELISA in 3.5 and 8 month-old conventionally-raised (CONVR-APPPS1) and germ-free APPPS1 mice (GF-APPPS1). Levels of Aβ assessed by western blot in 3.5 month-old (n = 5) **(e)** and in 8 month-old mice (n = 6). Full blots are shown in Supplementary Fig. 6 **(f)**. Plasmatic levels of soluble Aβ40 **(g)**, Aβ42 **(h)** and ratio Aβ42/Aβ40 **(i)** by ELISA. Experiments were performed three times, and data represent mean values of a representative experiment. Data represent mean ± SEM. Statistical differences between germ free and conventionally-raised mice: *p < 0.05, **p < 0.01, ***p < 0.001.

We first assessed levels of cerebral human Aβ38, Aβ40 and Aβ42 by ELISA. In both GF- and CONVR-APPPS1 mice Aβ42 exceeded Aβ40 by several-fold, whereas Aβ38 was barely detected (Fig. 2a–c). In both GF- and CONVR-APPPS1 mice, all three Aβ species showed a significant increase from 3.5 to 8 months of age (Aβ38: p < 0.001, Aβ40: p < 0.01, Aβ42: p < 0.01). However, in comparison to CONVR-APPPS1 mice, cerebral Aβ42 in GF-APPPS1 mice was significantly lower in both 3.5 and 8 month-old animals (−42%, p < 0.01 and −31%, p < 0.01, respectively). Aβ40 levels were also decreased but did not reach statistical significance (p = 0.12 and p = 0.15, respectively) (Fig. 2b–d). Consistent with the ELISA results, western blot assessment also demonstrated significantly lower Aβ levels in both 3.5 and 8 month-old GF-APPPS1 mice compared to CONVR-APPPS1 animals (−57%, p < 0.001 and 70%, p < 0.001, respectively) (Fig. 2e,f). Interestingly, at least in the aged GF-APPPS1 mice, cerebral APP levels were significantly higher compared to CONVR-APPPS1 animals (+24%, p < 0.05) (Fig. 2e,f). We then assessed plasma Aβ levels in GF- and CONVR-APPPS1 animals. While plasma Aβ42 levels were significantly decreased in young GF-APPPS1 mice compared to CONVR-APPPS1 mice (−29%, p < 0.01), plasma levels of Aβ40 and Aβ42 in aged animals remained unaffected (Fig. 2g–i).

### Aβ deposition in brain is reduced in GF-APPPS1 mice compared to CONVR-APPPS1 mice

**Figure 3:**
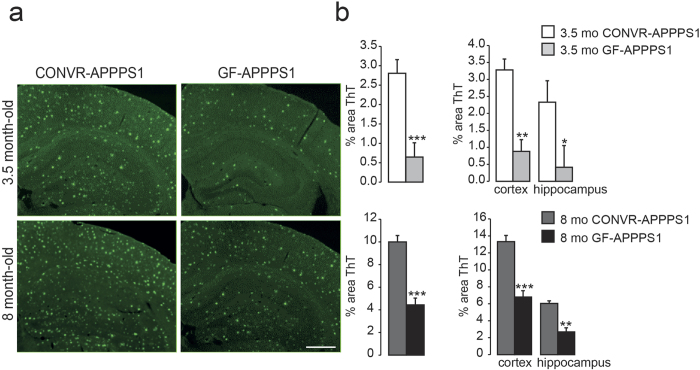
Reduced amyloid load in GF-APPPS1 transgenic mice. **(a)** Thioflavin T-stained brain sections encompassing the cortex and hippocampus of a 3.5 and 8 month-old CONVR-APPPS1 and age-matched GF-APPPS1 mice (scale bar: 150 um, all panels have the same magnification). **(b)** Quantification of amyloid load on Thioflavin-stained sections demonstrates a significant reduction of cerebral amyloid plaques in young and aged GF-APPPS1 mice (n = 5 and n = 6 respectively) in both cortex and hippocampus, as compared to CONVR-APPPS1 mice (n = 5 and n = 6 respectively). Statistical differences between GF- and CONVR-APPPS1 mice: *p < 0.05, **p < 0.01, ***p < 0.001. Shown are mean ± SEM.

Histopathological assessment was performed to evaluate cerebral Aβ amyloid load in GF- and CONVR-APPPS1 animals. Brain sections from APPPS1 animals were stained with Thioflavin T dye (Fig. 3a). Quantification of Thioflavin T-stained brain sections revealed a significant reduction of compact (Thioflavin-positive) Aβ amyloid load in both young and aged GF-APPPS1 brains (−77%, p < 0.001 and −57%, p < 0.001, respectively) as compared to age-matched CONVR-APPPS1 brains and this was true for the cortex (−73%, p < 0.01 and −55%, p < 0.001, respectively) and hippocampus (−82%, p < 0.05 and −60%, p < 0.01, respectively) (Fig. 3b). These results demonstrate that compact Aβ deposits were significantly reduced in the absence of gut microbiota in GF-APPPS1 animals. Reduction of Aβ pathology in GF- versus CONVR-APPPS1 animals was also confirmed qualitatively by immunostaining with the anti-Aβ antibody 6E10 (data not shown).

### Neuroinflammation changes in GF-APPPS1 transgenic mice

The reduction of cerebral Aβ amyloid load in GF-APPPS1 mice was accompanied by an overall decrease in cortical neuroinflammation, as evaluated by immunostaining with an antibody against the microglia marker Iba-1 (Fig. S3a). This observation was confirmed by quantitative imaging and revealed that both young and aged GF-APPPS1 animals exhibited a 64% and 40% decrease in Iba-1 positive immunostaining, respectively (p < 0.001 for both groups) as compared to the 3.5 and 8 month-old CONVR-APPPS1 control mice (Fig. S3b). Of note, immunostaining with only secondary anti-mouse antibody on GF-APPPS1 and CONVR-APPPS1 sections revealed no IgG leakage into the brain as evaluation of blood-brain-barrier leakage (data not shown). To assess the neuroinflammatory profile, cytokines were measured in total brain homogenates (Fig. S3c). As expected, the pro-inflammatory cytokine interleukin (IL)-1β increased with age in CONV-APPPS1 mice by 117% (p < 0.001). In contrast, IL-1β showed no significant increase with age in GF-APPPS1 mice (p = 0.25) but was 36% lower in 8 month-old GF mice compared to CONV-APPPS1 animals (p < 0.001). Interestingly, three T cell-associated cytokines, IFN-γ, IL-2 and IL-5, were also significantly altered. IFN-γ, IL-2 and IL-5 levels were highest in 3.5 month-old CONV-APPPS1 mice and were significantly decreased by 46% (p < 0.01), 44% (p < 0.01) and 54% (p < 0.05), respectively, in age-matched GF-APPPS1 mice.

### Increased levels of Aβ-degrading enzymes in GF-APPPS1 mice

To provide further insights into the mechanisms by which Aβ amyloid pathology was decreased in GF-APPPS1 animals, we assessed the levels of two Aβ degrading enzymes, neprilysin degrading enzyme (NPE) and insulin degrading enzyme (IDE)^16^,^17^. Indeed, as compared to age-matched CONVR-APPPS1 mice, levels of NPE were increased in both young and aged GF-APPPS1 animals by 116% and 209%, respectively (p < 0.01 and p < 0.001). Levels of IDE were increased in young GF-APPPS1 animals by 56% (p < 0.01), but similar in aged GF-APPPS1 mice (p = 0.43), as revealed by western blot analysis using an anti-IDE antibody (Fig. S4). We also investigated levels of APPCTF however no difference was found by western blot in young GF-APPPS1 animals as compared to age-matched CONVR-APPPS1 mice (p = 0.08). In contrast, levels of APP-CTF were increased in aged GF-APPPS1 animals APP-CTF (p < 0.05) (Fig. S4).

### Colonization of GF-APPPS1 mice with microbiota increases Aβ pathology

**Figure 4:**
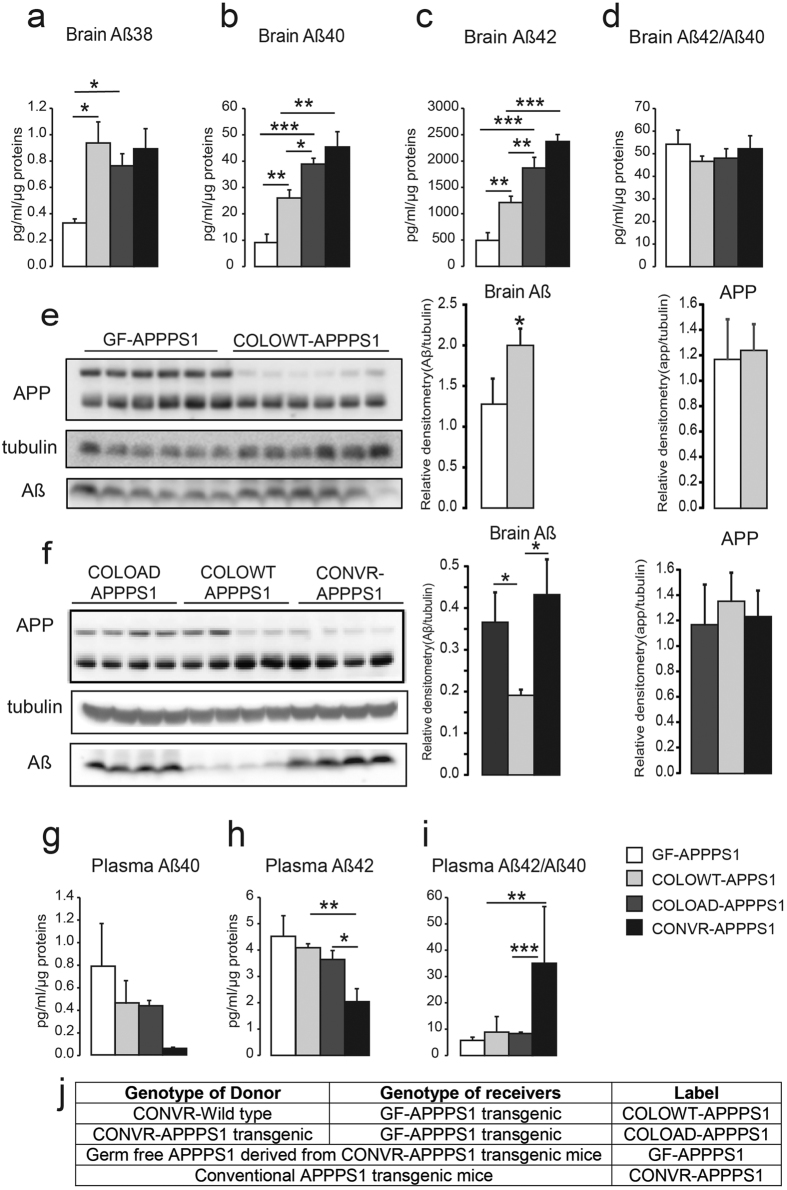
Colonization of 4 month-old GF-APPPS1 mice with the microbiota from aged CONVR-APPPS1 (COLOAD-APPPS1) and CONVR-wild type mice (COLOWT-APPPS1). After 8 weeks of colonization, levels of cerebral soluble Aβ38 **(a)**, Aβ40 **(b)**, Aβ42 **(c)** and ratio Aβ42/Aβ40 **(d)** were evaluated by ELISA in (6 month-old) GF-APPPS1, COLOWT-APPPS1, COLOAD-APPPS1 and CONVR-APPPS1 mice. Levels of Aβ assessed by western blot in 6 month-old GF-APPPS1 and COLOWT-APPPS1 mice (n = 6) **(e)** and in 6 month-old COLOAD-APPPS1, COLOWT-APPPS1 animals and CONVR-APPPS1 (n = 4) **(f)** Full blots are shown in Supplementary Fig. 7. Plasmatic levels of soluble Aβ40 **(g)**, Aβ42 **(h)** and ratio Aβ42/Aβ40 **(i)** by ELISA. Description of used genotypes **(j)**. Experiments were performed three times, and data represent mean values of a representative experiment. Data represent mean ± SEM. Statistical differences between germ free and conventionally-raised mice: *p < 0.05, **p < 0.01, ***p < 0.001.

To confirm a significant influence microbiota in the development of cerebral Aβ amyloidosis, 4 month-old GF-APPPS1 mice were colonized of gut microbiota from aged (12 month-old) CONVR-WT or CONVR-APPPS1 mice by oral gavage and were kept in a conventional environment for 8 weeks (COLOWT-APPPS1 and COLOAD-APPPS1 mice). ELISA revealed that soluble Aβ38 levels were increased respectively by 158% and 130% in COLOWT-APPPS1 and COLOAPPPS1-APPPS1 mice when compared to control GF-APPPS1 mice (p < 0.05), whereas levels of Aβ40 were increased by 184% and 326% in both COLOWT-APPPS1 and COLOAD-APPPS1, respectively (p < 0.01) (Fig. 4a,b). Levels of Aβ42 in COLOWT-APPPS1 mice were increased by 145% compared to control GF-APPPS1 mice (p < 0.01), without however reaching Aβ42 levels of COLOAD-APPPS1 mice (p < 0.01) or CONVR-APPPS1 mice (p < 001). Levels of Aβ42 in COLOAD-APPPS1 mice were increased by 278% compared to control GF-APPPS1 mice (p < 0.001), and did not differ significantly from Aβ42 levels in CONVR-APPPS1 (p = 0.22). Strikingly, levels of Aβ42 in COLOAD-APPPS1 mice were 54% higher than that observed in COLOWT-APPPS1 mice (p < 0.01) (Fig. 4c). Cerebral Aβ42/Aβ40 ratio was similar between the groups (Fig. 4d). Consistent with the ELISA results, western blot demonstrated that in contrast and in comparison to microbiota from aged CONVR-APPPS1 mice, control colonization of APPPS1 animals with microbiota from aged wild-type mice (COLOWT-APPPS1) was surprisingly less effective in increasing cerebral Aβ levels (Fig. 4e,f). APP levels remained unaffected between the groups. While plasma Aβ40 levels were similar between all groups, plasma Aβ42 levels were decreased in CONVR-APPPS1 mice when compared with COLOWT- and COLOAD-APPPS1 mice (p < 0.05). Aβ42/Aβ40 ratios were similar between GF-APPPS1, COLOWT- and COLOAD-APPPS1 mice but significantly increased in CONVR-APPPS1 group when compared with COLOWT- and COLOAD-APPPS1 mice (p < 0.01) (Fig. 4g–i). Albeit not significant, we also observed a trend toward a decrease in enzymatic activity of NPE and IDE in colonized mice, indicating that NPE and IDE plays a role in Aβ degradation in germ free APPPS1 mice (Fig. S4).

### Gut microbiota composition in GF-APPPS1 mice colonized with microbiota from CONVR-APPPS1 and CONVR-WT mice

**Figure 5:**
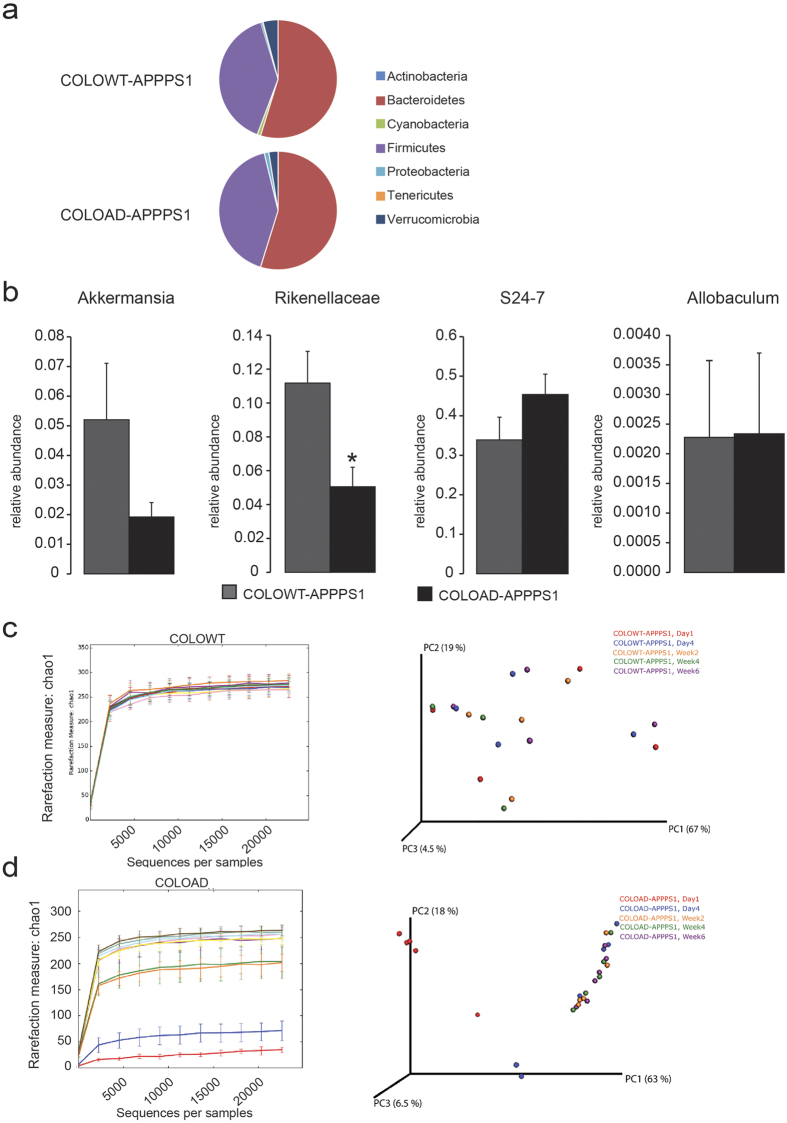
Comparison of the gut microbiota between COLOWT-APPPS1 and COLOAD-APPPS1 mice at 6 weeks after colonization. **(a)** Mean sequence relative abundance of gut microbial taxa at phylum level in COLOWT-APPPS1 and COLOAD-APPPS1 mice (n = 6 and n = 6 respectively). No significant differences were observed at this time point. **(b)** At genus level, the 4 bacterial taxa that differed in the aged CONVR-APPPS1 and CONVR-WT mice showed similar trends regarding *Akkermansia* abundance in the colonized mice, but did not reach statistical significance. Experiments were performed three times, and data represent mean values of a representative experiment. Data represent mean ± SEM. Rarefaction curves (α-diversity vs. sequencing effort) and weighted Unifrac PCoA plot to compare phylogenetic distance matrices of **(c)** COLOWT-APPPS1 and **(d)** COLOAD APPPS1 mice at day 1, day 4 and week 2, 4 and 6 of colonization. Significant differences in α-diversity were found between COLOAD-APPPS1 and COLOWT-APPPS1 mice at Day 1 (p < 0.05) as well as between COLOAD-APPPS1 at Day 1 and Day 4, Week 2, 4 and 6 (all p < 0.05).

Comparison of microbiota in COLOAD-APPPS1 and COLOWT-APPPS1 mice revealed that, at phylum level, microbiota from aged CONVR-APPPS1 mice lowered the abundance of Verrucomicrobia (Fig. 5a) at week 6 of colonization. However, at genus level, although a trend toward decrease was observed, *Akkermansia* abundance was not significantly lower in COLOAD**-**APPPS1 mice (Fig. 5b). In contrast, significant differences in bacterial taxa between COLOAD-APPPS1 and COLOWT-APPPS1 mice were found at earlier time points of colonization, including lower abundance of *Rikenellaceae, Ruminococcus*, S24-7 (p < 0.001) and *Dorea* (p < 0.01) in COLOAD-APPPS1 mice at day 1 of colonization (data not shown). At day 4, COLOAD-APPPS1 mice had lower RF32 and at week 2 lower abundance of *Bacteroides* (p < 0.001). Correspondingly, mice colonized with microbiota from CONVR-APPPS1 mice appeared to have a slower development of their microbial diversity when compared with mice colonized with microbiota from CONVR-WT mice, as the wild type-colonized mice had a high diversity already at day 1 of colonization (Fig. 5c,d). Furthermore, in the aged CONVR-APPPS1 and colonized mice, the relative abundance of 8 bacterial genera were correlated with the amount of cerebral soluble Aß42 (p < 0.05, after corrected for multiple comparisons), including *Parabacteroides, Akkermansia, Pseudomonas, Odoribacter, Anaerofustis,* unclassified genera in *Mogibacteriaceae* and *Xanthomonadaceae* families and an unclassified family in *RF39* order (Fig. S5).

## Discussion

Increasing evidence suggests the gastro-intestinal tract is the bridge between microbiota and the central nervous system^18^,^19^,^20^. While the link between the microbiome and brain disorders is starting to emerge^18^,^19^,^20^ the impact of gastro-intestinal microbes on the development of AD is not known and has also not adequately been addressed in model system despite often anecdotal evidence that amyloid plaque formation in APP transgenic mice differs among mouse facilities with different specific-pathogen-free (SPF) conditions^21^,^22^.

Indeed, the present finding of marked differences in the gut microbiota composition between aged APPPS1 mice and age-matched wildtype control mice suggests that distinct microbial constitutions can influence the development of cerebral β-amyloidosis. We found that the abundance of at least two major phyla (Firmicutes and Bacteroidetes) in the fecal microbiota was significantly altered in APPPS1 mice. At genus level, *Allobaculum* and *Akkermansia* were decreased and unclassified genera of *Rikenellaceae* and *S24*-*7* increased. Of note the control mice were littermates and were housed in the same cage as the APPPS1 mice. It is a common approach to co-house wild type and transgenic mice to minimize other confounding factors (e.g. cage-effects)^23^. The genotype is a strong influencer of the gut microbiome and as a first approach, we aimed to characterize the microbiome in APPPS1 transgenic mice, using a standardized approach of co-housing to minimize other influences. Reduced levels of *Akkermansia* have been associated with obesity and type 2 diabetes in mice^24^, and prebiotic-induced restoration of *Akkermansia* in the gut results in reduced fat-mass gain and decreased systemic inflammation. In turn, type 2 diabetes and inflammation are known risk factors in AD development^25^,^26^,^27^,^28^. Strikingly, the relative abundance of *Akkermansia* was negatively correlated and several bacterial genera were positively correlated with the amount of the pathogenic Aß42 in brain.

Our colonization experiments with microbiota from APPPS1 mice resulted in similar differences in *Akkermansia* and *S24*-*7*, along with increased AD-like pathology compared to colonization with microbiota from WT mice, indicating that these two microbial taxa may influence progression of AD pathology. *Akkermansia* can increase gut barrier integrity and thus control metabolic endotoxemia^24^. One can hypothesize that depleted in *Akkermansia* in AD mice may lead to a disturbed gut barrier with increased influx of inflammatory components including endotoxins. Consistent with this hypothesis, Kumar *et al*. showed that gastro-intestinal microbiota of the brains of transgenic 5XFAD mice resulted in seeding and accelerated cerebral beta-amyloid deposition^29^. The family of *S24*-*7* bacteria in the *Bacteroidales* order have been linked to autoimmune diabetes and probiotic administration in mice can reduce disease together with decreased *S24*-*7* abundance^30^. As *Parabacteroides*, also a member of the *Bacteroidales* order, was negatively correlated with AD-like pathology, our results suggest that a shift in *Bacteroidales* alleviate, from possibly protective *Parabacteroides* to *S24*-*7* may affect cerebral Abeta amyloid progression. Taken together, it is possible that enriched and depleted microbial taxa observed in AD mice affect gut immune cell signaling; taxa observed in WT mice instead have a positive impact on e.g. regulatory T-cells in the gut, which have been shown to down-regulate neuroinflammatory signaling in a mouse model of experimental autoimmune encephalomyelitis^31^. Higher diversity of gut microbiota has been observed in lean individuals when comparing to obese individuals, but diversity appears to be a complex parameter to interpret, as some more recent microbiota studies instead have shown higher diversity in disease states, such as in coeliac disease^32^ and colorectal cancer^33^. It was recently observed that prebiotics reduced atherosclerosis and low-grade systemic inflammation in mouse models, together with altered microbiota composition and lower diversity of bacteria^34^. Thus, rather than simply counting the number of bacterial species in the gut, a more comprehensive analysis of enriched and depleted microbial taxa must be performed and diversity alterations defined for each disease state where the microbiota has a proven role. The altered gut microbial flora in the aged APPPS1 mice was not observed to the same extent in the 1 month-old and 3.5 month-old APPPS1 mice. However, the gradual increase in the microbiota shift with age is a further indication that gut bacteria might be involved in the progression of Aß lesion in brain. This conclusion is in line with the present colonization study in which harvested microbiota from aged APPPS1 mice dramatically increase the levels of cerebral Aβ in germ-free APPPS1 mice. Remarkably, in the brain pathogenicity of microbiota harvested from aged wild-type control mice was less pronounced compared to the microbiota harvested from aged APPPS1 mice that further hints to putative pathogenic gastro-intestinal microbiota strain(s) in the APPPS1 mouse model.

This view is consistent with the observation that the absence of intestinal microbiota in the GF-APPPS1 transgenic model was sufficient to significantly decrease cerebral Aβ amyloid pathology. Both biochemical levels of Aβ and the extent of compact Aβ plaques were consistently decreased in the brains of APPPS1 transgenic mice without gut microbiota. Interestingly, the positive impact of the absence of microbiota on the Aβ lesions was already significant in the 3.5 month-old GF-APPPS1 animals. The observation of similar or even higher APP levels in GF-APPPS1 mice compared to age-matched, CONVR-APPPS1 animals strongly implies that the decrease of Aβ amyloid pathology in the GF-APPPS1 mice is not due to lower APP expression, but rather results from a mechanism occurring downstream of APP production and cleavage. While our data on plasmatic Aβ42 levels in young GF-APPPS1 animals indicate that clearance of cerebral Aβ to the periphery is increased, the absence of such an effect in aged GF-APPPS1 animals suggests that increased efflux of Aβ from the brain does not significantly account for decreased Aβ amyloid pathology. Although in our preclinical experiments the microbiota appears to regulate Abeta amyloid deposition, this may not ultimately affect AD pathogenesis as the human disease is now recognized as multifactorial^35^. Of note, the APPPS1 mouse model used in our study does not display robust behavioral abnormalities at least in young animals, and thus does not permit to unraveling potential behavioral changes in the absence of gastro-intestinal microbiota.

Remarkably, we observed significant alterations in the brain’s immune response in GF-APPPS1 mice. Our results showing reduced microgliosis and changes in the brain cytokine profile are in line with a recent publication demonstrating that germ-free mice show immature microglia and reduced pro-inflammatory cytokine production^36^. Importantly, caspase-1 knockout (which prevents the production of IL-1β) has been shown to be sufficient to strongly reduce plaque load in APPPS1 animals through altering the microglial activation state and enhancing microglial phagocytosis of Aβ plaques^37^. Therefore, a change in microglial responses in germ-free APPPS1 animals could contribute to the reduction of amyloid load observed in germ-free animals. Several *in vitro* and *in vivo* studies have shown that NPE and IDE can degrade Aβ. Most notably, NPE and IDE levels were increased in GF-APPPS1 mice, indicating that increased levels of these Aβ degrading enzymes may contribute to decreased cerebral Aβ amyloidosis in germ free animals. Despite a strong trend toward an increase, similar levels of APP-CTF in young animals indicated that activity of gamma-secretase does not significantly account in differences observed in young GF- versus CONV-APPPS1 animals. However, the significant increase in APP-CTF in aged GF-APPPS1 suggest that gamma secretase activity might be altered in GF APPPS1 mice. Altogether, these results indicate that Aß degrading enzymes may partially play a role in decreasing Aβ levels and cerebral Aß amyloidosis in germ free animals.

Our pre-clinical results using an axenic mouse model of AD demonstrate that the absence of microbiota retards substantially the progression of AD-like pathology. The association of bacterial taxa with cerebral Aβ pathology observed in conventionally raised APPPS1 mice indicates that specific microbes may be involved in progression of cerebral Abeta amyloidosis. The gut colonization studies underline the importance of the nature of the donor from which microbiota is harvested (e.g., diseased transgenic mice versus WT mouse model) for the promotion of AD. Thus, our study strongly argues for a role of gastro-intestinal microbes in the development of cerebral Aβ amyloidosis. Obviously, the clinical translation of these preclinical results bears the potential for opening a new area for the treatment and prevention of AD pathology and ultimately other neurodegenerative disorders. Our results further support the emerging view that microbiota contribute to the development of a wide range of neurological and neurodegenerative diseases well beyond metabolic syndrome, diabetes and obesity.

## Methods

### APPPS1 mice

APPPS1 transgenic mice were maintained at the Ecole Polytechnique Fédérale de Lausanne animal core specific pathogen free facility. There was unlimited access to autoclaved food and water. APPPS1 animals co-express the KM670/671NL Swedish mutation of human amyloid precursor protein (APP) and the L166P mutation of human presenilin 1 (PS1) under the control of the Thy-1 promoter, and show age-dependent accumulation of parenchymal Aβ plaques with minimal vascular Aβ amyloid that is restricted to the pial vessels^14^. APPPS1 mice were generated on a C57BL/6 background. Both male and female APPPS1 mice as well as age-matched control wild-type (WT) littermates were used. APPPS1 mice and wildtype littermate were housed together in grouped cages (n = 5 mice/cage) until analyzed. Throughout the study these mice are referred to as conventionally-raised (CONVR) APPPS1 and WT mice.

### Approval of animal studies

Animal studies were performed according to regulations issued by the Swiss government and approved by the ethic veterinary committee of the cantons of Vaud and Bern, Switzerland.

### APPPS1 germ-free mice

For germ-free rederivation, 4–6 week-old APPPS1 transgenic female mice were superovulated by intraperitoneal injection of 5 IU pregnant mare serum gonadotropin (PMSG, VWR) on day 0, and 5 IU of human chorionic gonadotropin (hCG) on day 2. Females were paired with APPPS1 males on day 2. Two days later, plugged females were euthanized, oviducts collected, and the embryos were flushed out of the oviducts. The fertilized 2-cell embryos were collected and extensively washed in M2 medium (Sigma) containing Penicillin/Streptomycin (Invitrogen). Subsequently these embryos were transferred into pseudo-pregnant germ-free NMRI recipient females under aseptic conditions. Resulting litters were maintained in flexible film isolators at the Clean Mouse Facility at the University of Bern with unlimited access to autoclaved food and water. Both male and female GF-APPPS1 mice as well as age-matched control wild-type littermates (GF-WT) were used. Note that GF-APPPS1 and GF-WT mice were fed with the same autoclaved diet (Kliba-Nafag PN3437) as was used for the CONVR-APPPS1 and CONVR-WT mice (see above). Moreover GF-APPPS1 and GF-WT mice were housed in grouped cages (n = 5 mice/cage), were handled 2 times a week exactly the same as CONVR-APPPS1 and CONVR-WT mice (see above). Due to the complexity of the experimental design (generation of germ free animals), no statistical methods could be used to predetermine sample size. Furthermore the experiments were not randomized and the investigators were not blinded to allocation during experiments and outcome assessment.

### Bacteriological status of the GF-APPPS1 model

Mice were regularly checked for germ-free status by aerobic and anaerobic culture, DNA staining (Sytox-green, Invitrogen) and gram staining of cecal content to detect uncultivable contamination. In addition, serological testing for known viruses and pathogens was performed periodically by ELISA, PCR or IFA. The obtained negative results confirm the germ free status of our animals (Tables S1 and S2).

### Preparation of transgenic samples for analyses

All mice were prepared at the EPFL, i.e. GF-APPPS1 mice and GF-WT mice raised in Bern were shipped to EPFL in sterile containers 24 hours prior to analysis. On the day of sacrifice, mice were weighed and euthanized by lethal intraperitoneal injection of pentobarbital (150 mg/kg; <200 mg/ml); stomachs, livers, pancreas and brains were collected, weighed and snap frozen in liquid nitrogen for further analysis. Brains were extracted and cut in half; one half was immediately incubated in 4% paraformaldehyde in ice-cold PBS and used for histology (see below); the other hemisphere was snap frozen and used for biochemical analysis (ELISA and western blot; see below). Blood was collected from the veina cava, transferred to Heparin coated tubes, centrifuged 4′500 rpm 4 °C for 15 min. Plasma was transferred to fresh clean tubes and snap frozen for further analysis.

### Histology and immunohistochemistry

After 2 days fixation in 4% paraformaldehyde (PFA), the half brains were incubated for 48 h in 30% sucrose. Brains were then frozen in 2-propanol (Merck) and subsequently sectioned on a freezing-sliding microtome to collect 25 μm coronal sections. For histological staining, brain sections were stained with Thioflavin T dye according to standard protocol (ThT 1% in 50% Ethanol; Sigma Aldrich, St. Louis, MO). Sections were immunostained to visualize Aβ deposits using mouse monoclonal antibody (6E10, 1:500; COVANCE). Microglial reaction was assessed using a rabbit polyclonal antibody to Iba-1 (1:1000; Wako, 019–19141). Vectastain Elite ABC Kits were used and revelation obtained with Vector SG Blue (Vector Laboratories).

### Quantification of Thioflavin T-positive amyloid load

Hemi-brain sections encompassing the cortex and hippocampus, and stained for Thioflavin T (ThT), were quantified for compact amyloid load. To acquire images used for quantification, ThT-stained brain sections were imaged with a 10x objective using a Zeiss Axiovert 200 M/ApoTome microscope (Zeiss, Germany) coupled with a Zeiss Axiocam HR camera (Zeiss, Germany). For each of the animals involved in this study, 10 ± 2 stained coronal brain secions with a thickness of 25 μm and spaced from each other by 24 slices were available for imaging. Among these, four sections were selected based on the following criteria: one section encompassing the cortex (between position AP 0.74 mm and AP 0.38 mm from Bregma), one section with the striatum (between the position AP - 0.46 mm and AP - 0.70 mm from Bregma), one section showing the dorsal hippocampus (between position AP - 1.82 mm and AP - 2.18 mm from Bregma) and one section encompassing the ventral hippocampus (between position AP - 2.70 mm and AP - 3.08 mm from Bregma). For each selected section, 1300 × 1030 contiguous images (with less than 5% overlapping) were captured for the entire cortex and hippocampus region (when present). The acquired images were analyzed with the public domain software Image J. Analyzed areas were adjusted manually so that each measurement was accurately measuring only the region of interest and adjusted to exclude brains regions other than the cortex and hippocampus. For ThT-load analysis, the images were inverted and a threshold of approximately 180 was applied (with a 5% variance to best adjust tissue staining variations among sections). ThT-stained brain sections were analyzed with parameters of larger than 5 × 5 pixels and with a circularity of 0.15.

### Quantification of Iba-1-positive immunoreactivity

Neocortical microglia immunoreactivy was quantified in hemi-brain sections immunostained for activated microglia (with the Iba-1 antibody). Acquisition of images from Iba-1-stained sections was similar to that described for ThT quantification. Subsequently, a threshold of 110 with a variance of 10% was applied to each image using Image J. This threshold was selected in order to best display the microglia while minimizing the background noise. After visual inspection, ROI were selected such that staining artefacts on the section was manually removed by cropping. The microglia were quantified using the analyze particles tool embedded in ImageJ. In order to consider both the single microglia and the microglial loads, the pixel size was not specified and the circularity parameter was set to 0.0–1.0.

### Statistical analysis (Histology)

Data represent the means ± standard errors of the means. Statistical analysis was performed using ANOVA followed by Hom-Sidak’s test for multiple comparisons and unpaired t test followed by Welch’s correction.

### Western blot and biochemistry

Brains were extracted and homogenized in RIPA buffer (150 mM sodium chloride, 1.0% NP-40 or Triton X-100, 0.5% sodium deoxycholate, 0.1% sodium dodecyl sulfate, 50 mM Tris, pH 8.0) (10% weight/volume). Brain homogenates were centrifuged at 14’000 rpm for 15 min at 4 °C. The purified protein fractions were stored at −80 °C until western blotting. Total protein concentration of each sample was determined using a BCA protein assay kit (Pierce, Rockford, IL, USA). For Western blot analysis, 25 μg of protein was loaded into a precast 15-well NuPAGE Novex 12% Bis-Tris gel (Invitrogen, Waltham, MA) for separation by electrophoresis and then transferred to a polyvinylidenedifluoride (PVDF) membrane as indicated in the manufacturer’s instructions (GE Healthcare, UK). As primary antibodies, mouse monoclonal anti Aβ antibody (6E10, Covance), mouse monoclonal anti-ß tubulin antibody (cell signaling) were incubated at 4 °C overnight, followed by secondary horseradish peroxidase-anti-mouse antibody (Jackson Labs, Baltimore) incubation. The blots were visualized with BM chemiluminescence Western blot kit (Roche, Basel).

Neprilysin (NPE) and Insulin degrading enzymes (IDE) activity: NPE and IDE activity have been measured, according to the manufacturer instructions with fluorometric SensoLyte® 520 Neprilysin Activity Assay Kit (AS-72223, ANASPEC) and SensoLyte® 520 IDE Activity Assay Kit (AS-72231, ANASPEC) respectively.

### Statistical analysis (Western Blots)

Graphpad Prism 6 software was used to perform statistical analysis. Data represent the means ± standard errors of the means. Statistical analysis was performed using ANOVA followed by Hom-Sidak’s test for multiple comparisons and unpaired t test followed by Welch’s correction.

### ELISA

Brain protein extracts and plasma were diluted to the fourth into sampling medium provided by the manufacturer and a final of 25 μl volume was loaded into a 96 well plates. Aβ38, Aβ40 and Aβ42 measurements were performed according to the manufacturer’s instructions (Peptide Panel 1 (6E10) Kit (1 Plate) V-PLEX™K15200E-1 Mesoscale Discovery, Gaithersburg). All levels of Aβ38, Aβ40 and Aβ42 were normalized against total protein amount. For cytokine measurements, total brain homogenates were centrifuged at 14’000 rpm, 30 min, 4 °C and supernatants were analyzed using the mouse pro-inflammatory panel 1 V-plex plate (Mesoscale Discovery) according to the manufacturer’s instructions and normalized against total protein content.

### Statistical analysis (ELISA)

Graphpad Prism 6 software was used to perform statistical analysis. Data represent the means ± standard errors of the means. Statistical analysis was performed using ANOVA followed by Hom-Sidak’s test for multiple comparisons.

### Colonization study

Cecal contents were pooled from amyloid-depositing (12 month-old) CONVR-APPPS1 mice or CONVR-WT mice. Cecal contents were homogenized in sterile PBS (2 ml per cecum) and a volume of 0.2 ml was immediately administered by oral gavage to 4 month-old littermate GF-APPPS1 mice, as previously described^38^. Oral gavage was given at day 1 and day 4. The resulting transplanted mice (termed as COLOAD-APPPS1 and COLOWT-APPPS1) were housed in a conventional environment for 8 weeks under the same conditions and fed with the same diet as their CONVR-APPPS1 counterparts. Control colonization study was performed using cecal contents pooled from control wild-type (12 month-old) mice and given intraorally to GF-APPPS1 mice (COLOWT-APPPS1). During feces collection, mice were put in clean litter-free individual cages until stool collection. Fresh feces were collected from each mouse at day 1 and 4 and at week 2, 4 and 6 for bacterial 16S rRNA gene sequencing. Mice were analysed 8 weeks after the beginning of the colonization.

### DNA extraction

One frozen faecal pellet/mouse was thawed on ice and DNA was extracted using the QIA amp DNA Stool Mini Kit (Qiagen). The protocol from the manufacturer was followed with an addition of a bead beating step. Sterile glass beads (1 mm) were added together with stool lysis buffer to the samples and cell disruption was performed for 2 × 2 minutes at 25 Hz using a TissueLyser (Qiagen), followed by a heating step at 95 °C for 5 minutes. After lysis, DNA-damaging substances and PCR inhibitors were removed using InhibitEX tablet (provided with the kit) and the DNA was purified on QIAamp Mini spin columns.

### PCR amplification of the V3–4 region of bacterial 16 S rRNA genes

16S rRNA genes were amplified by PCR with forward and reverse primers containing Illumina adapter sequences and unique dual indexes used to tag each PCR product^39^, according to the 16S-protocol provided by Illumina. Briefly, PCR reactions were carried out in 25-μL reactions with 0.2 μM forward and reverse primers, with 12.5 ng template DNA and 12.5 μl of 2 × KAPA HiFi HotStart Ready Mix kit (KAPA Biosystems). Thermal cycling consisted of initial denaturation at 95 °C for 3 min followed by 25 cycles of denaturation at 95 °C for 30 s, annealing at 55 °C for 30 s, and extension at 72 °C for 30 s, followed by a final step of 72 °C for 5 min. The amplicons (464 bp product) were purified with Agencourt AMPureXP Kit (Beckman Coulter). A second PCR was thereafter performed to attach Illumina adapters and unique dual indexes to each sample, followed by a clean-up step with AmPureXP Kit (Beckman Coulter) (Table S3). PCR amplicons were visualized using 0.1% agarose gel electrophoresis. Negative extraction controls did not produce visible bands.

### Amplicons quantitation, pooling, and sequencing

Mouse fecal amplicon DNA concentrations were quantified using the Qubit3.0 Fluorometer (Life Technologies, Stockholm, Sweden). Amplicons were combined in equimolar ratios into a single tube with a final concentration of 4 pM. As an internal control, 5% of PhiX was added to the amplicon pool. Paired-end sequencing with a read length of 2 × 300 bp was carried out on a Miseq Instrument (Illumina) using a Miseq reagent kit v3 (Illumina Inc., San Diego, USA).

### Sequence analysis

Sequences were analysed with the free software package Quantitative Insights into Microbial Ecology (QIIME), which allows analysis of high-throughput community sequencing data. Default values were used for each step, except where otherwise specified^40^. Sequences were removed if lengths were <200 nt, contained ambiguous bases, primer mismatches or homo polymer runs in excess of six bases. Forward and reverse reads were joined using Fastqjoin. After quality filtering, a total of 6,571,748 sequence reads were generated for the 8 month-old mice (APPPS1 n = 6, WT n = 7), 5,713,081 reads for the 3.5 month-old mice (APPPS1 n = 7, WT n = 8) and 3′688′179 reads for the 1 month-old mice (APPPS1 n = 6, WT n = 8) with an average number of 505,519 reads per sample for the 8 month-old mice, 361,568 reads/sample for the 3.5 month-old and 263,411 reads/sample for the 1 month-old mice. For the colonized mice, a total of 5,550,818 sequence reads was generated after quality filtering with an average of 79,297 reads per sample. Similar sequences were binned into operational taxonomic units (OTUs) using UCLUST^41^, with a minimum pairwise identity of 97%, generating 1369 OTUs for the CONVR-APPPS1 and WT mice, and 1,837 OTUs for the COLO-mice. The most abundant sequence in each OTU was chosen to represent its OTU. Representative sequences from each OTU were aligned using PyNAST (a python-based implementation of NAST in QIIME)^39^ and taxonomy was assigned using the Greengenes database (v. 13_8)^42^ and the RDP classifier^43^.

### Statistical analysis (metagenomics)

Graphpad Prism 6 software was used to identify significant differences in bacterial relative abundances between transgenic CONVR-APPPS1 and WT mice using multiple t-tests and the Holm-Sidak method of correction for multiple comparisons, at phylum and genus level. To find correlations between brain Aβ42 levels and bacterial genera in the aged and COLO-APPPS1 mice, the operational taxonomic units (OTU) tables were rarefied at 291,712 and 22,609 randomly selected sequences/sample, respectively, for the entire data set after which orthogonal partial least squares (OPLS) scatter plot analysis was performed using SIMCA14 (Umetrics, Umeå, Sweden). Pearson’s correlation was calculated for each pairwise combination of brain Aβ42 and bacterial genera using Graphpad Prism 6 and the p-values were then corrected by Benjamin-Hochberg procedure for multiple comparisons^44^. Further, α- and β-diversities were analyzed in QIIME on rarefied data, using 291,712 sequences/sample for 8 month-old mice, 178,620 for 3.5 month-old mice, 136,829 for 1 month-old mice, and 22,609 for mice colonized with APPPS1 and WT microbiota. Differences in α-diversity were calculated in QIIME using a non-parametric t-test and FDR correction for multiple comparisons and β-diversity differences were analyzed with the ANOSIM and Adonis non-parametric statistical tests in QIIME. Linear discriminant analysis effect size (LEfSE, www.huttenhower.sph.harvard.edu/galaxy/) was applied on the OTU table according to ref. 45.

## Additional Information

**How to cite this article:** Harach, T. *et al*. Reduction of Abeta amyloid pathology in APPPS1 transgenic mice in the absence of gut microbiota. *Sci. Rep.* **7**, 41802; doi: 10.1038/srep41802 (2017).

**Publisher's note:** Springer Nature remains neutral with regard to jurisdictional claims in published maps and institutional affiliations.

## Supplementary Material

Supplementary Information
