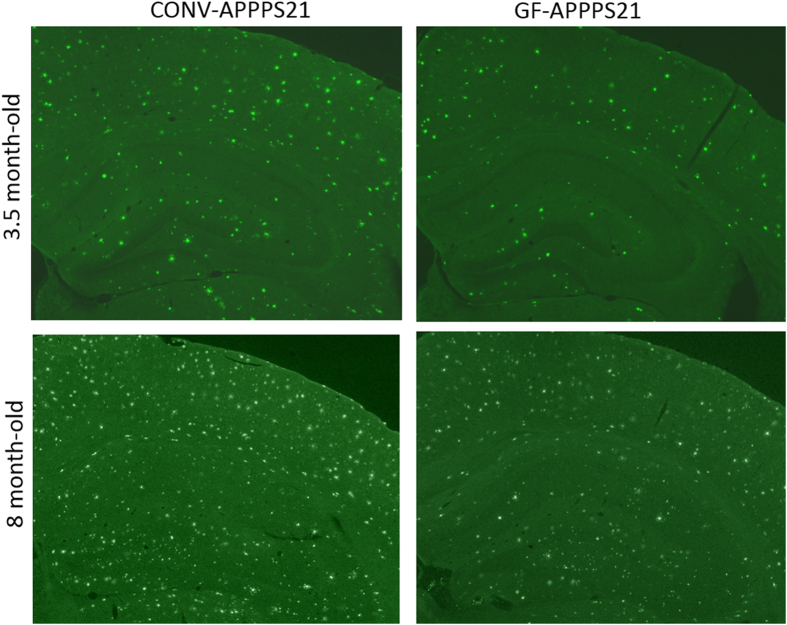# Erratum: Reduction of Abeta amyloid pathology in APPPS1 transgenic mice in the absence of gut microbiota

**DOI:** 10.1038/srep46856

**Published:** 2017-07-10

**Authors:** T. Harach, N. Marungruang, N. Duthilleul, V. Cheatham, K. D. Mc Coy, G. Frisoni, J. J. Neher, F. Fåk, M. Jucker, T. Lasser, T. Bolmont

Scientific Reports 7: Article number: 41802; 10.1038/srep41802 published online: 02 08 2017; updated: 07 10 2017.

This Article contains errors in the Discussion section.

“Our colonization experiments with microbiota from APPPS1 mice resulted in similar differences in Akkermansia and S24-7, along with increased AD-like pathology compared to colonization with microbiota from WT mice, indicating that these two microbial taxa may influence progression of AD pathology. Akkermansia can increase gut barrier integrity and thus control metabolic endotoxemia24. One can hypothesize that depleted in Akkermansia in AD mice may lead to a disturbed gut barrier with increased influx of inflammatory components including endotoxins. Consistent with this hypothesis, Kumar *et al*. showed that gastro-intestinal microbiota of the brains of transgenic 5XFAD mice resulted in seeding and accelerated cerebral beta-amyloid deposition29. The family of S24-7 bacteria in the Bacteroidales order have been linked to autoimmune diabetes and probiotic administration in mice can reduce disease together with decreased S24-7 abundance30. As Parabacteroides, also a member of the Bacteroidales order, was negatively correlated with AD-like pathology, our results suggest that a shift in Bacteroidales alleviate, from possibly protective Parabacteroides to S24-7 may affect cerebral Abeta amyloid progression”.

should read:

“Our colonization experiments with microbiota from APPPS1 mice resulted in similar differences in Akkermansia and S24-7, along with increased AD-like pathology compared to colonization with microbiota from WT mice, indicating that these two microbial taxa may influence progression of AD pathology. Akkermansia can increase gut barrier integrity and thus control metabolic endotoxemia24. One can hypothesize that gastro-intestinal microbiota depleted in Akkermansia in AD mice may lead to a disturbed gut barrier with increased influx of inflammatory components including endotoxins. Consistent with this hypothesis, Kumar *et al*. showed that bacterial infection of transgenic 5XFAD mice resulted in seeding and accelerated cerebral beta-amyloid deposition29. The family of S24-7 bacteria in the Bacteroidales order have been linked to autoimmune diabetes and probiotic administration in mice can alleviate disease together with decreased S24-7 abundance30. As Parabacteroides, also a member of the Bacteroidales order, was negatively correlated with AD-like pathology, our results suggest that a shift in Bacteroidales composition, from possibly protective Parabacteroides to S24-7 may affect cerebral Abeta amyloid progression”.

“Remarkably, in the brain pathogenicity of microbiota harvested from aged wild-type control mice was less pronounced compared to the microbiota harvested from aged APPPS1 mice that further hints to putative pathogenic gastro-intestinal microbiota strain(s) in the APPPS1 mouse model”.

should read:

“Remarkably, pathogenicity of microbiota harvested from aged wild-type control mice was less pronounced compared to the microbiota harvested from aged APPPS1 mice that further hints to putative pathogenic gastro-intestinal microbiota strain(s) in the APPPS1 mouse model”.

In addition, Figure 3 has duplicated panels for CONVR-APPPS1 at 3.5 month-old and GF-APPPS1 at 8 month-old. The correct Figure 3 appears below as Figure 1.

## Figures and Tables

**Figure 1:**