# Supplementary material for: Reduction of Abeta amyloid pathology in APPPS1 transgenic mice in the absence of gut microbiota
Source: Sci Rep. 2017 Feb 8;7:41802. doi: 10.1038/srep41802 (PMC5297247; doi:10.1038/srep41802)
Supplement: Supplementary Information [file srep41802-s1.doc]

TITLE

Reduction of Abeta amyloid pathology in APPPS1 transgenic mice

in the absence of gut microbiota

**Authors:** T. Harach^1^, N. Marungruang^2^, N. Dutilleul^1^, V. Cheatham^1^, K. D. Mc Coy^3^, G. Frisoni^4^, J. J. Neher^5^, F. Fåk^2^, M. Jucker^5^, T. Lasser^1^, and T. Bolmont^1^

**Affiliations:**

^1^ Laboratoire d'Optique biomédicale, Institute of Microengineering, School of Engineering, Ecole Polytechnique Fédérale de Lausanne, CH1015 Lausanne, Switzerland.

^2^ Food for Health Science Centre, Lund University, Medicon Village, 22381 Lund, Sweden.

^3^ Mucosal Immunology Lab, Department of Clinical Research, University of Bern, Murtenstrasse, 35 CH - 3010 Bern, Switzerland.

^4^ Memory Clinic and LANVIE - Laboratory of Neuroimaging of Aging, University Hospitals and University of Geneva, Geneva, Switzerland.

^5^ German Centre for Neurodegenerative Diseases (DZNE), Tübingen, D-72076 Tübingen, Germany & Department of Cellular Neurology, Hertie Institute for Clinical Brain Research, University of Tübingen, D-72076 Tübingen, Germany.

**SUPPLEMENTAL FIGURE LEGENDS**

**Fig. s1.** Comparison of the gut microbiota between 8 month-old transgenic (CONVR-APPPS1) and wild type (CONVR-WT) mice. **(a)** Cladogram generated with LDA Effect Size (LEfSE) analysis (α=0.01) displays bacterial taxa with LDA score higher than 2, illustrating gut microbial taxa present in higher abundance in 8 month-old CONVR-APPPS1(red) and CONVR-WT (green) mice (n= 6 and n= 7 respectively). Size of circles is proportionate to each taxon’s mean relative abundance. **(b)** Body weight, cecum weight and food intake in 8 month-old CONVR-APPPS1 and CONVR-WT mice (n= 6 and n= 7 respectively). Data represent mean ± SEM. The only statistical difference was found for cecum weight (p< 0.05).

**Fig. s2**. Correlation between the gut microbiota and brain Aβ levels in 8 month-old CONVR-APPPS1 mice (n=6). (**a**) Orthogonal partial least squares (OPLS) scatter plot giving an overview of the correlations. Variables situated closed to each other were positively correlated and variables situated opposite each other were negatively correlated. Ten bacterial genera were positively correlated with Aß42 levels (p< 0.05, Pearson’s correlation with adjusted p-values for multiple comparisons using Benjamini-Hochberg procedure). (**b**) Scatter plot of the ten significant correlations with determination coefficient (R2) indicating how well the data fits the model.

**Fig. s3**. Decreased neuroinflammation in GF-APPPS1 mice. Representative sections immunostained with Iba-1 in young (GF-APPPS1, n=5 and CONV-APPPS1, n=5) and aged CONVR-APPPS1 mice (n=6) (left panels) and GF-APPPS1 mice (right panels) (n=6) (scale bar: 150 um, all panels have the same magnification) **(a)**. Quantification of Iba-1 immunoreactivity demonstrates a significant reduction of activated neocortical microglia in GF-APPPS1 mice as compared to CONVR-APPPS1 animals. Statistical differences between GF- and CONVR-APPPS1 mice: *: p< 0.05, **: p< 0.01, ***: p< 0.001. Shown are mean ± SEM **(b)**. Cytokine profiles in GF-APPPS1 and CONVR-APPPS1 mice. 3.5 month-old GF-APPPS1 mice (n=5) show significantly reduced levels of interferon (IFN)-γ, interleukin (IL)-2 and IL-5 compared to CONR-APPPS1 animals. Further, while there is a significant increase of IL-1β with age in CONVR-APPPS1 mice, IL-1β levels are significantly lower in 8 month-old GF animals (n=6). Other cytokines, namely IL-10, IL-12p70, IL-4, IL-6, KC/GRO and TNF-α show no significant alterations in GF compared to CONVR-APPPS1 mice. Experiments were performed three times, and data represent mean values of a representative experiment. Data represent mean ± SEM. Statistical differences between GF-APPPS1 mice and CONV-APPPS1 transgenic mice: * p< 0.05, ** p< 0.01, *** p< 0.001 **(c)**.

**Fig. s4**. Levels of Neprilysin (NPE), Insulin degrading enzyme (IDE) and APP-CTF in the brain of 3.5 (**a**) and 8 month-old (**b**) GF-APPPS1 and CONR-APPPS1 mice. Levels of NPE were increased in young and aged GF-APPPS1 animals compared to age-matched CONVRAPPPS1 mice. Levels of IDE were increased in young GF-APPPS1 animals compared to age matched CONVR-APPPS1 mice, but similar between aged animals. Levels of APP-CTF did not differ significantly between young animals but were increased in aged GF-APPPS1 animals compared to age-matched CONV-APPPS1 mice. (**c**) Cerebral enzymatic activity of Neprilysin (NPE) and Insulin degrading enzyme (IDE) in GF-APPPS1, COLOWT-APPPS1 and COLOAD-APPPS1.

**Fig. s5.** Correlation between the gut microbiota and cerebral soluble Aβ42 in 8 month-old CONVR-APPPS1 (n=6), COLOAD-APPPS1 (n=6) and COLOWT-APPPS1 (n=6) mice. Orthogonal partial least squares (OPLS) scatter plot giving an overview of the correlations. Variables situated closed to each other were positively correlated and variables situated opposite each other were negatively correlated. Microbial genera in green are those significantly correlate with brain Aß42 levels **(a).** Two and six bacterial genera were positively and negatively correlated with Aß42 levels, respectively (p< 0.05, Pearson’s correlation). Scatter plot of the eight significant correlations with determination coefficient (R2) indicating how well the data fits the model **(b).**

**Fig. s6**. Non-cropped blots. Levels of Aβ assessed by western blot in 3.5 month-old CONVR-APPPS1 and GF-APPPS1 mice (n= 5) and in 8 month-old old CONVR-APPPS1 and GF-APPPS1 mice (n= 6)

**Fig. s7**. Non-cropped blots. Levels of Aβ assessed by western blot in 6 month-old GF-APPPS1 and COLOWT-APPPS1 mice (n= 6) **(e)** and in 6 month-old COLOAD-APPPS1, COLOWT-APPPS1 animals and CONVR-APPPS1 (n= 4).

**Fig. s8**. Non-cropped blots. Levels of Insulin degrading enzyme (IDE) and APP-CTF in the brain of 3.5 (a) and 8 month-old (b) GF-APPPS1 and CONR-APPPS1 mice.

**SUPPLEMENTAL FIGURES**

**
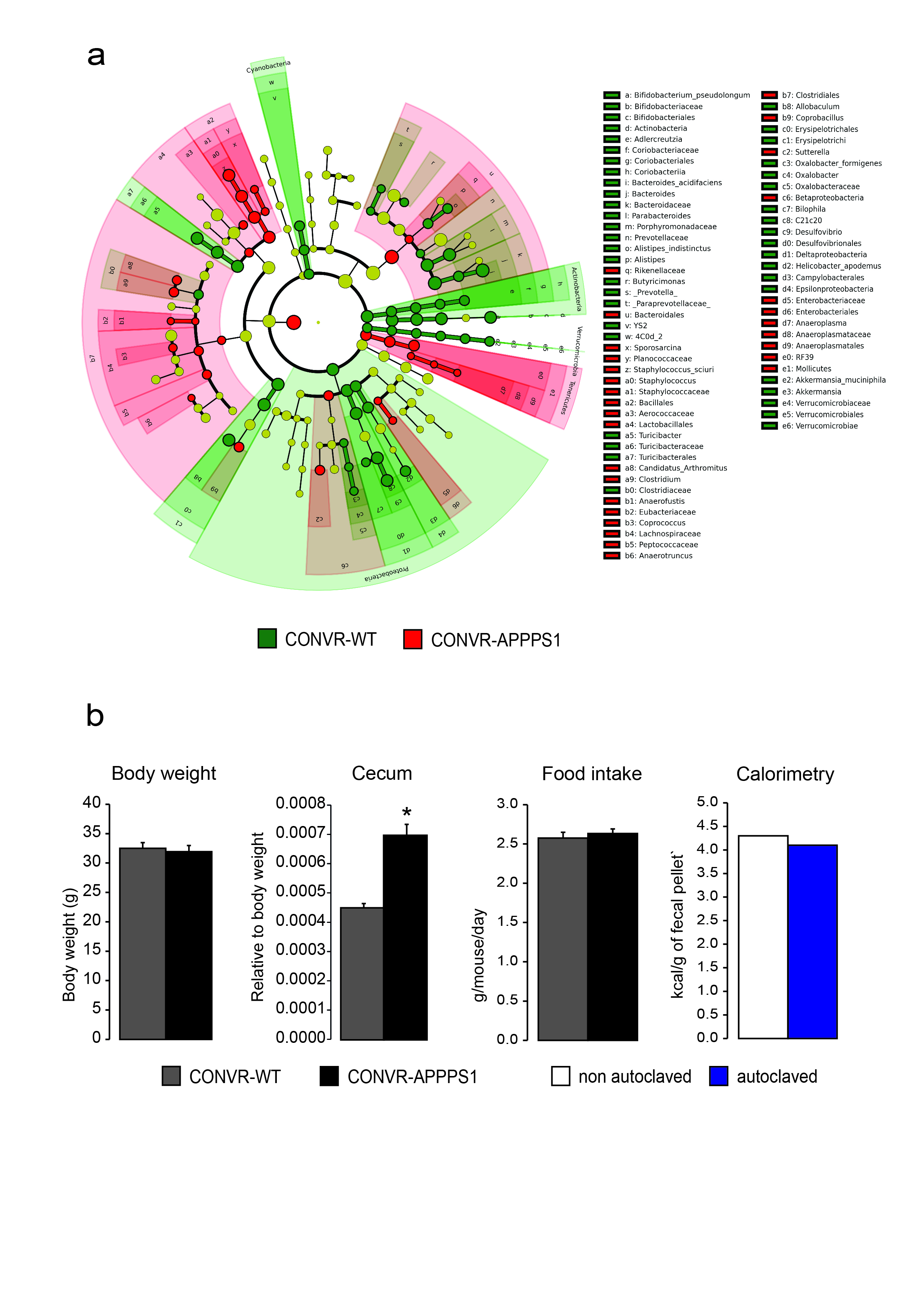
**

**Fig. s1**


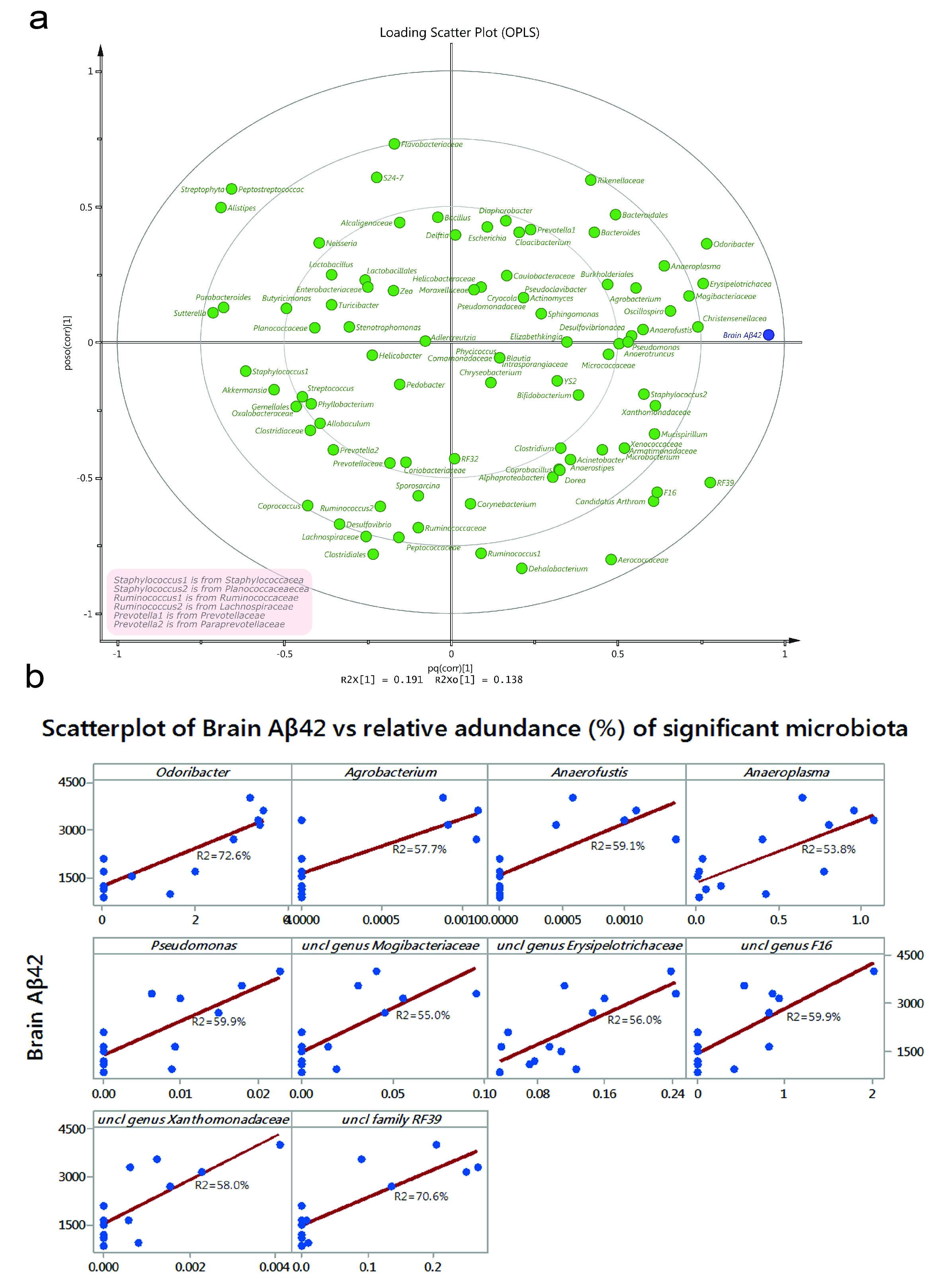


**Fig. s2**

**
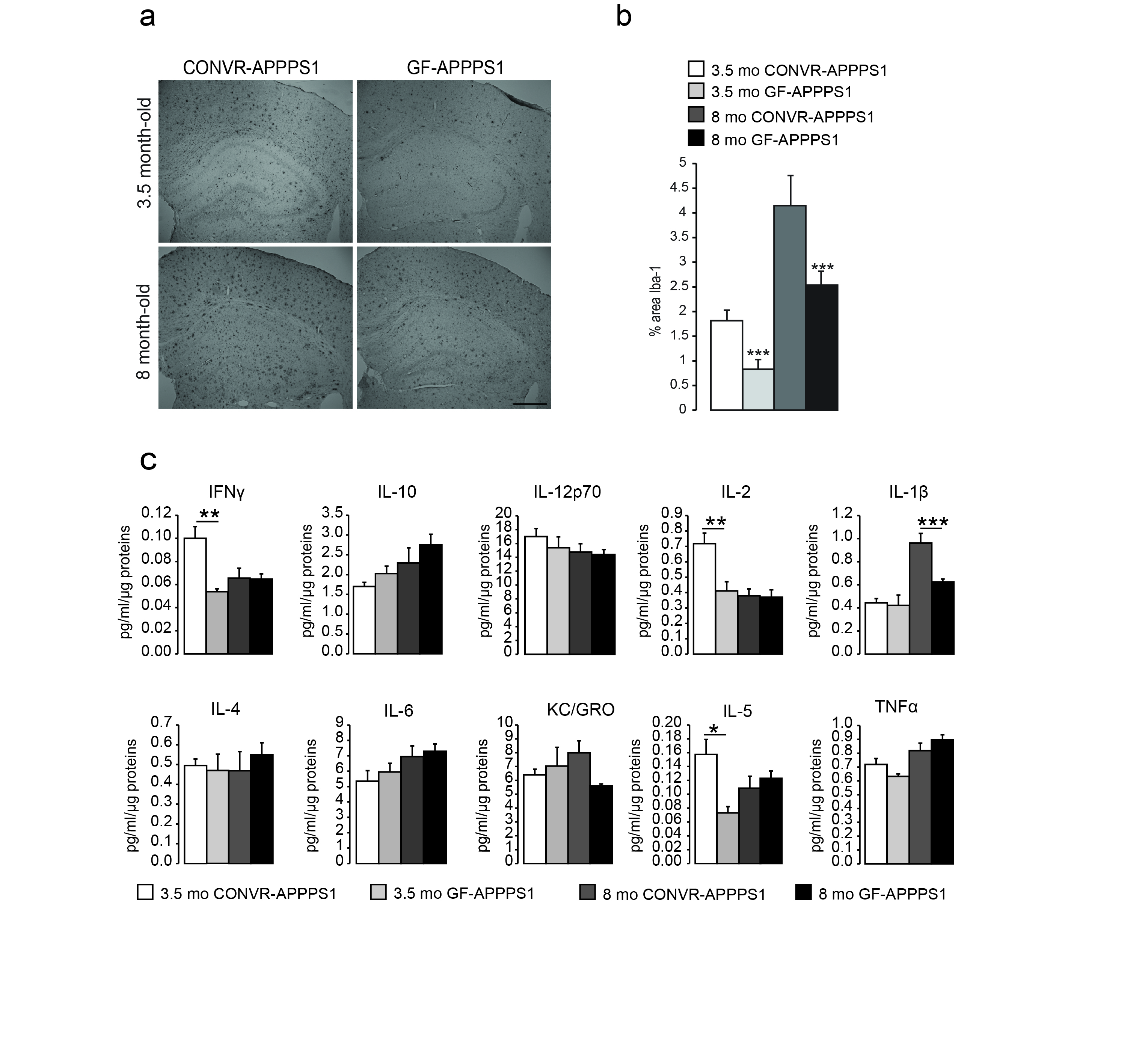
**

**Fig. s3**


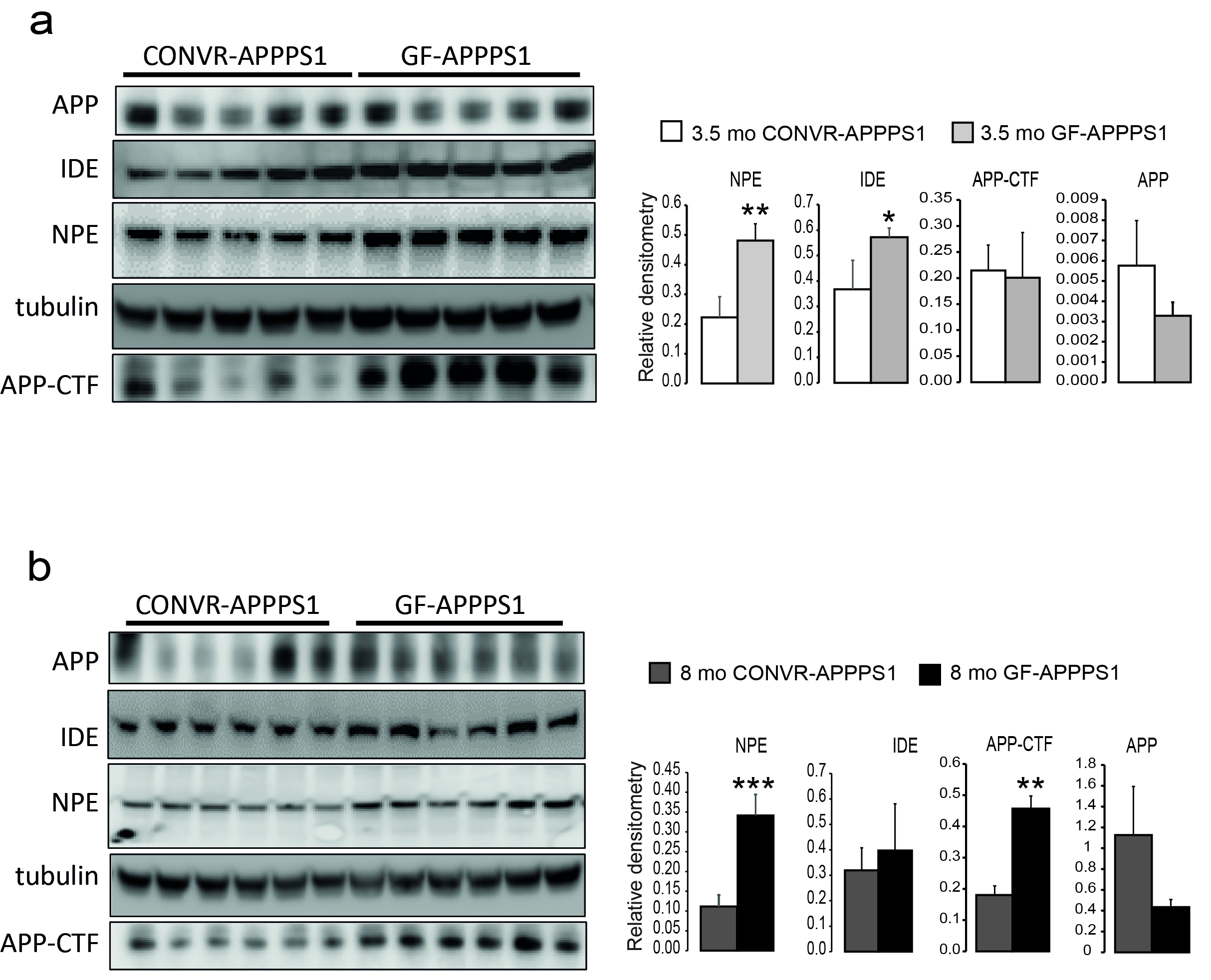
­


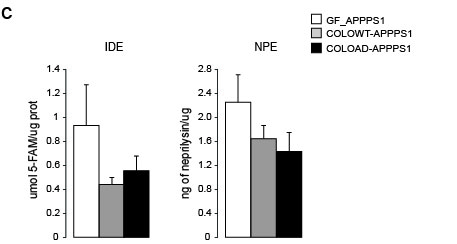


**Fig. s4**

**
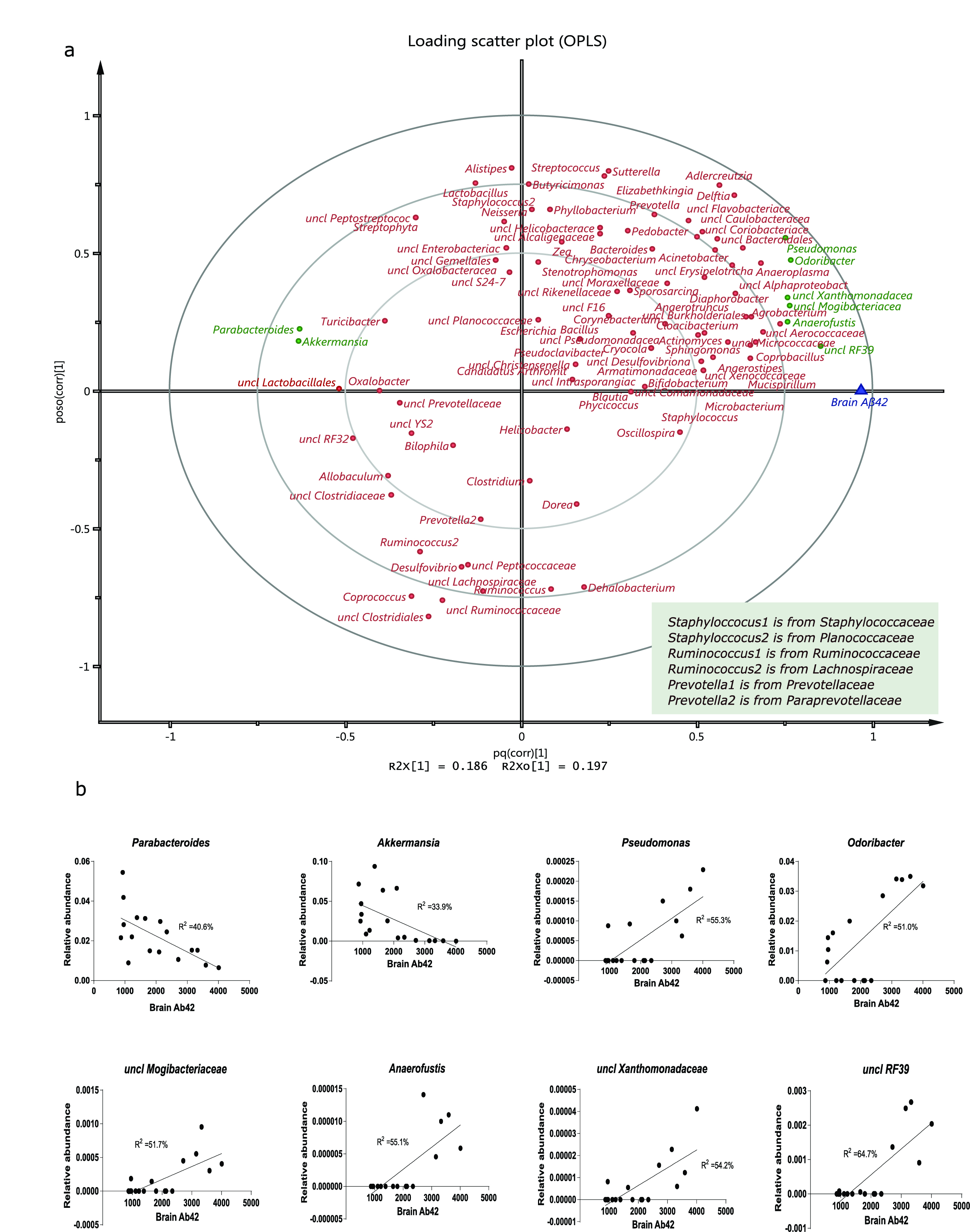
**

**Fig. s5**


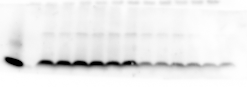

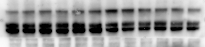

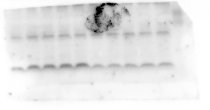

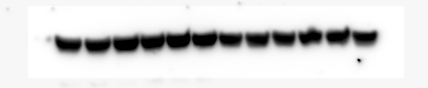

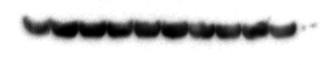

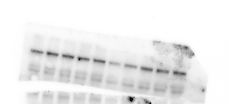


**APPPS1_CONV**

**APPPS1_GF**

Aβ

Tubulin

APP

**APPPS1_CONV**

**APPPS1_GF**

4Kd

50Kd

100Kd

4Kd

100Kd

50Kd

**Fig. s6**

**­­**


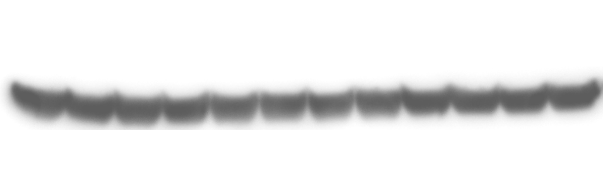

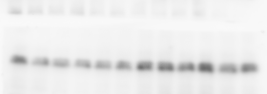

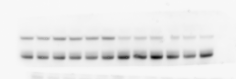

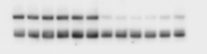

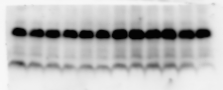

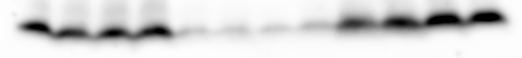


**COLOWT_APPPS1**

**APPPS1_GF**

**COLOAD APPS1**

**COLOWT APPS1**

**CONVR**

**APPS1**

Aβ

Tubulin

APP

Aβ

Tubulin

APP

4Kd

50Kd

100Kd

4Kd

50Kd

100Kd

**­**

**Fig. s7**

**
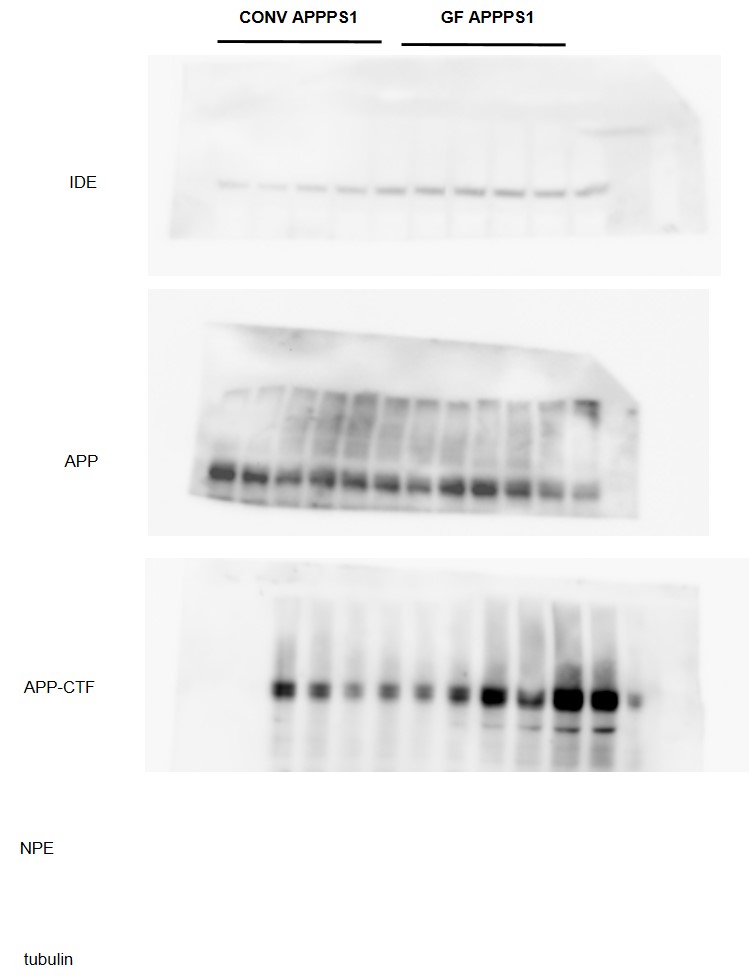
**

**Fig. s8**

**SUPPLEMENTAL TABLES**

|  |  | Aerobic culture  ceacal content | Anaerobic culture ceacal content | Gram stain ceacal content | Cytox DNA stain ceacal content |
| --- | --- | --- | --- | --- | --- |
| 8.1.2014 | Isolator BE.09 Sentinel #1 | neg | neg | neg | neg |
| 8.1.2014 | Isolator BE.09 Sentinel #2 | neg | neg | neg | neg |
| 7.2.2014 | Isolator BE.09 Sentinel #1 | neg | neg | neg | neg |
| 7.2.2014 | Isolator BE.09 Sentinel #2 | neg | neg | neg | neg |
| 8.4.2014 | Isolator BE.09 Sentinel #1 | neg | neg | neg | neg |
| 8.4.2014 | Isolator BE.09 Sentinel #2 | neg | neg | neg | neg |

**Table S1.** Aerobic, anaerobic culture, sytox-green and gram staining of germ free cecal content. “neg” refers to negative detection at mentioned time points.

| ***Viruses*** | | | | |
| --- | --- | --- | --- | --- |
| Ectromelia virus | Serum | IFA | 0/2 | 0/2 |
| Lymphocyticchoriomeningitis (LCMV) | Serum | ELISA | 0/2 | 0/2 |
| Mouse adenovirus, FL | Serum | ELISA | 0/2 | 0/2 |
| Mouse adenovirus, K87 | Serum | ELISA | 0/2 | 0/2 |
| Mouse cytomegalovirus (MCMV) | Serum | IFA | 0/2 | 0/2 |
| Mouse Rotavirus (EDIM) | Serum | IFA | 0/2 | 0/2 |
| Mouse hepatitis virus (MHV) | Serum | nPCR | 0/2 | 0/2 |
| MurineNorovirus (MNV) | Serum | IFA | 0/2 | 0/2 |
| Parvoviruses: Mouse parvovirus (MPV) | Serum | IFA | 0/2 | 0/2 |
| Parvoviruses: minute virus of mice (PVM) | Serum | IFA | 0/2 | 0/2 |
| Reovirus type 3 (REO 3) | Serum | IFA | 0/2 | 0/2 |
| Sendai virus | Serum | IFA | 0/2 | 0/2 |
| Theiler'smurineencephalomyelitis (TMEV) | Serum | IFA | 0/2 | 0/2 |
| Theiler (GDVII) | Serum | IFA | 0/2 | 0/2 |
| ***Mycoplasma*** | | | | |
| Mycoplasma pulmonis | Serum | IFA | 0/2 | 0/2 |
| ***Bacteria*** | | | | |
| Citrobacter rodentium | RLGImt* | Culture | 0/2 |  |
| Clostridium piliforme | Serum | IFA | 0/2 | 0/2 |
| Corynebacterium kutscheri | RLGImt* | Culture |  | 0/2 |
| Helicobacter sp. | Caecum | nPCR | 0/2 |  |
| Pasteureillaceae | RLGImt* | Culture | 0/2 |  |
| Salmonella spp. | RLGImt* | Culture | 0/2 |  |
| Streptoba caillusmoniliformis | RLGImt* | Culture | 0/2 |  |
| Streptococcus pneumoniae | RLGImt* | Culture | 0/2 |  |
| beta-haemolytische Streptococcen | RLGImt* | Culture | 0/2 |  |
| Bordetella bronchiseptica | RLGImt* | Culture | 0/2 |  |
| Klebsiella oxytoca | RLGImt* | Culture | 0/2 |  |
| Staphylococcus aureus | RLGImt* | Culture | 0/2 |  |
| Pseudomonas sp. | RLGImt* | Culture | 0/2 |  |
| Pseudomonas aeruginosa | RLGImt* | Culture | 0/2 |  |
| Yersiniapseudo tuberculosis | RLGImt* | Culture | 0/2 |  |
| Pasteurellapneumotropica | Serum | IFA | 0/2 | 0/2 |
| ***Parasites*** | | | | |
| Ecotoparasites | Skin/Pelt | Microscopy | 0/2 |  |
| Endoparasites-Protozoa | Native test | Microscopy | 0/2 |  |
| Helminths | Caecum | Microscopy | 0/2 |  |
| Toxoplasmagondii | Serum | IFA | 0/2 |  |
| *RLGImt* : Respiratory Tract/Lung,GI Tract, external mucosa, throat*  *nPCR: PCR non GLP* | | | | |

**Table S2.** Serological testing for common viruses and pathogens.

| **16S Amplicon PCR** Forward Primerw | 5'TCGTCGGCAGCGTCAGATGTGTATAAGAGACAGCCTACGGGNGGCWGCAG |
| --- | --- |
| **16S Amplicon PCR** Reverse Primer | 5'GTCTCGTGGGCTCGGAGATGTGTATAAGAGACAGGACTACHVGGGTATCTAATCC |
| **Illumina Forward** overhang | 5’ TCGTCGGCAGCGTCAGATGTGTATAAGAGACAG‐16S-V3-4-specific sequence |
| **Illumina Reverse** overhang | 5’ GTCTCGTGGGCTCGGAGATGTGTATAAGAGACAG‐16S-V3-4-specific sequence |

**Table S3.** Primer sequences for amplification and sequencing of 16S rRNA genes.
